# Macrophage inhibitory cytokine 1 (MIC-1/GDF15) as a novel diagnostic serum biomarker in pancreatic ductal adenocarcinoma

**DOI:** 10.1186/1471-2407-14-578

**Published:** 2014-08-08

**Authors:** Xiaobing Wang, Yanfen Li, Haimei Tian, Jun Qi, Mo Li, Chao Fu, Fan Wu, Yi Wang, Dongwan Cheng, Wenya Zhao, Chao Zhang, Teng Wang, Jianyu Rao, Wei Zhang

**Affiliations:** Medical Center for Tumor Detection, Cancer Institute and Hospital, Chinese Academy of Medical Sciences and Peking Union Medical College, Beijing, 100021 PR China; Laboratory of Clinical Biochemistry, Cancer Institute and Hospital, Chinese Academy of Medical Sciences and Peking Union Medical College, Beijing, 100021 PR China; Laboratory of Clinical Biochemistry, Xi’an NO 4 hospital, Xi’an, 710004 PR China; Department of Abdominal Surgical Oncology, Cancer Institute and Hospital, Chinese Academy of Medical Sciences and Peking Union Medical College, Beijing, 100021 PR China; Department of VIP, Cancer Institute and Hospital, Chinese Academy of Medical Sciences and Peking Union Medical College, Beijing, 100021 PR China; National Laboratory of Biomacromolecules, Institute of Biophysics, Chinese Academy of Sciences, Beijing, 100021 PR China

## Abstract

**Background:**

Macrophage inhibitory cytokine 1 (MIC-1/GDF15) has been identified as a potential novel biomarker for detection of pancreatic cancer (PCa). However, the diagnostic value of serum MIC-1 for pancreatic ductal adenocarcinoma (PDAC), particularly for those at the early stage, and the value for treatment response monitoring have not yet been investigated.

**Methods:**

MIC-1 expression in tumor tissue was analyzed by RT-PCR from 64 patients with PDAC. Serum MIC-1 levels were detected by ELISA in 1472 participants including PDAC, benign pancreas tumor, chronic pancreatitis and normal controls. The diagnostic performance of MIC-1 was assessed and compared with CA19.9, CEA and CA242, and the value of it as a predictive indicator for therapeutic response and tumor recurrence was also evaluated.

**Results:**

MIC-1 levels were significantly elevated in PDAC tissues as well as serum samples. The sensitivity of serum MIC-1 for PDAC diagnosis was much higher than that of CA19.9 (65.8% vs. 53.3%) with similar specificities. Furthermore, serum MIC-1 detected 238 out of 377 (63.1%) CA19.9-negative PDAC. Moreover, receiver operating characteristic (ROC) curve analysis also showed that serum MIC-1 had a better performance compared with CA19.9 in distinguishing early-stage PDAC from normal serum with a higher sensitivity (62.5% vs. 25.0% respectively). Notably, serum MIC-1 level was significantly decreased in patients with PDAC after curative resection and returned to elevated levels when tumor relapse occurred.

**Conclusions:**

Serum MIC-1 is significantly elevated in most PDAC, including those with negative CA19.9 and early stage disease, and thus may serve as a novel diagnostic marker in early diagnosis and postoperative monitoring of PDAC.

## Background

Pancreatic ductal adenocarcinoma (PDAC) accounts for 95% of pancreatic cancer (PCa) and has a dismal prognosis, with only a 6% 5-year survival rate [1]. Owing to diagnostic and therapeutic progress over the past decades, the PDAC 5-year survival has been improved to 30-40% in about 15% patients who are eligible for potentially curative therapies at the time of diagnosis [2, 3]. Unfortunately, most of the patients with PDAC are diagnosed at an advanced stage due to the lack of obvious symptoms, and their prognosis remains very dismal [4, 5]. Thus, early detection and diagnosis of PDAC still present the best chance for successful treatments and improved outcomes.

CA19.9 has been widely used as a serologic diagnostic tumor marker for PDAC, and its usefulness and clinical significance have been reported in many studies [6, 7]. However, serum CA19.9 is elevated in less than 50% of early stage PDAC, and its efficacy for predicting prognosis and monitoring patients remains controversial. Many alternative biomarkers, such as CEA and CA242, have been investigated and used in clinical settings; but, their diagnostic value for early PDAC has been limited [6, 8–10]. Therefore, it is necessary to identify new serologic biomarkers with sufficient sensitivity to detect PDAC at an early stage and with potential for predicting prognosis and monitoring patients.

Macrophage inhibitory cytokine 1 (MIC-1/GDF15), a 25-kDa secreted growth factor of transforming growth factor-β (TGF-β) super-family, was originally discovered in macrophage cells [11, 12]. MIC-1 is weakly and stably expressed in most tissues under normal conditions, but is substantially upregulated under pathological conditions such as injury, inflammation and various cancers [13–17]. Considerable evidence has indicated that MIC-1 plays a significant role in carcinogenesis related activities, such as proliferation, migration, apoptosis, and angiogenesis, in many types of solid tumors including PDAC [18–28]. A previous study identified MIC-1 as a potential novel biomarker for detection of PCa [29–33]. However, the diagnostic value of serum MIC-1 for PDAC, particularly for those at the early stage, and the value for treatment response monitoring have not yet been investigated comprehensively, which is the aim of this study.

## Methods

### Study population and sample preparation

We collected 64 paired PDAC tissue samples (cancerous and matched adjacent normal tissues), which were verified by post-surgical pathological examination (cancer institute and hospital, Chinese Academy of Medical Sciences, Peking, China; CICAMS). Matched serum samples were also obtained to investigate the relationship between serum MIC-1 and tissue MIC-1 expression. All the patients have undergone surgery at CICAMS from 2001 to 2008. The clinicopathologic characteristics of these PDAC patients are summarized in Table 1. The corresponding normal tissues were obtained at least 2 cm away from the primary tumor.

**Table 1:** **Characteristics of subjects with PDAC and controls**

	Tissue samples	Serum samples in the discovery group				Serum samples (post-operative)		Serum samples	Serum samples in the validation group	
Variable	Cases (n= 64)	Healthy controls (n=500)	Benign disease (n=115)	Chronic pancreatitis (n=50)	PDAC cases (n=807)	Curative PDAC cases (n= 102)	Non-curative PDAC cases (n= 31)	PDAC cases (n= 35)	Healthy controls (n= 50)	Stage 1 PDAC cases (n= 50)
Gender (n)										
Male	35	287	63	27	438	57	17	19	28	31
Female	29	213	52	23	369	45	14	16	22	19
Age (years)										
≤45	23	102	26	17	111	30	4	8	15	16
46-55	25	153	34	14	215	38	4	13	12	14
56-65	14	121	29	12	272	23	9	8	12	13
>65	2	124	18	7	209	11	14	6	11	7
Stage (n)										
I	25				45	31		4		50
II	39				127	68	2	26		
III					337	3	29			
IV					298					

For serum samples, we recruited 1472 subjects in the discovery group and 100 subjects in the validation group. The clinicopathologic characteristics of the participants from above two groups are presented in Table 1. The discovery group included 807 PDAC and 115 benign pancreas tumors diagnosed between January 1, 2001 and December 31, 2010 (CICMAS, Peking, China), 50 chronic pancreatitis cases and 500 age- and gender-matched healthy subjects (by physical examination). The validation group included 50 stage I PDAC patients and 50 normal controls in the same hospital from December 2008 to November 2012. The samples from this independent validation group were not included in the discovery process and were evaluated in a blinded manner (the statistician had no prior information related to the samples) to avoid optimism in reporting performance. We also recruited an additional 240 cases with colorectal adenocarcinoma (n = 30), prostate adenocarcinoma (n = 30), gastric adenocarcinoma (n = 30), ovarian carcinoma (n = 30), breast carcinoma (n = 30), thyroid carcinoma (n = 30), esophageal squamous cell carcinoma (ESCC, n = 30) and non-small-cell lung carcinoma (NSCLC, n = 30), as diagnosed by post-surgical pathological examination (Table 2).

**Table 2:** **Characteristics of the subjects with eight types of epithelial malignancies (n = 240) in addition to PDAC and normal subjects**

Pathological feature	BC	TC	OC	ESCC	GA	PA	NSCLC	CA
Cases (n)	30	30	30	30	30	30	30	30
Gender (n)								
Male	0	14	0	17	16	30	15	18
Female	30	16	30	13	14	0	15	12
Age (years)								
≤45	11	11	7	5	2	3	6	4
46-55	12	14	17	9	13	8	12	9
55-65	6	3	5	11	11	9	8	10
>65	1	2	1	5	4	10	4	7
Stage (n)								
I	4	8	2	5	4	6	3	5
II	6	14	10	12	9	7	11	14
III	9	7	8	9	10	9	9	7
IV	11	1	10	4	7	8	7	4

BC: breast carcinoma; TC: thyroid carcinoma; OC: ovarian carcinoma; ESCC: esophageal squamous cell carcinoma; GA: gastric adenocarcinoma; PA: prostate adenocarcinoma; NSCLC: non-small-cell lung carcinoma; CA: colorectal adenocarcinoma.

Additionally, serum samples at one month post-surgery were collected from 102 of the 807 PDAC patients undergoing curative resection without inflammatory complications in the discovery group. Of the 102 cases, 35 patients with relapsed disease were included for monitoring the role of serum MIC-1 in response to curative resection and early recurrence. Meanwhile, serum samples at one month post-surgery were also collected from 31 patients undergoing noncurative resection.

None of the cases involved in our present study had undergone chemotherapy or radiotherapy prior to sampling, and subjects with inflammatory complications were also excluded from this project. The pathological evaluation was based on the criteria outlined by the American Joint Committee on Cancer staging criteria. This study has obtained human research ethics approval from the Ethics Committee of CICAMS.

### Quantification of MIC-1 mRNA by real-time quantitative RT-PCR

Total RNA of cancerous and matched normal tissues was extracted using TRIzol (Invitrogen) and assessed by measuring absorbance at 260 nm. Reverse transcription to synthesize the first strand of cDNA was performed with M-MLV reverse transcriptase (Promega). The resulting cDNA was then subjected to real-time quantitative PCR for the evaluation of the relative mRNA levels of MIC-1 and GAPDH (glyceraldehyde-3-phosphate dehydrogenase, as an internal control) with the following primers: MIC-1 forward: 5′-GGTGCTCATTC AAAAGACCGA-3′ and reverse: 5′-CATTCCACAGGGCAGGACA-3′.GAPDH forward: 5′-CTCCTCCTGT TCGACAGTCAGC-3′and reverse: 5′-CCCAATACGACCAAATCCGTT-3′. Gene- specific amplification was performed using an LightCycler 480 real-time PCR system (Life Technologies). The mix was preheated at 95°C (10 min), and amplified at 95°C (30 sec) and 55°C (1 min) for 45 cycles. The resolution curve was measured at 95°C for 15 sec, 55°C for 15 sec and 95°C for 15 sec. The Ct (threshold cycle) value of each sample was calculated from the threshold cycles with the instrument’s software, and the relative expression of MIC-1 mRNA was normalized to the GAPDH value.

### Quantification of MIC-1 and other biomarkers by immunoassay

Samples of venous blood were collected using the VACUETTE blood collection system. Blood was centrifuged for 10 minutes at 1700 × g. The serum was stored frozen at -80°C until use. Samples were thawed once just prior to analyses. We measured a panel of four markers, namely CEA, CA19.9, CA242 and MIC-1. Serum levels of MIC-1 were measured using a sensitive in house sandwich ELISA produced by CICAMS, of which the detection limit level was 20 pg/mL and the coefficient of variation was <10% [34]. Briefly, 50 μl various concentrations of standard recombinant MIC-1 and serum samples were added to each well of a 96-well plate that has been coated with 5 μg/ml of monoclonal anti-MIC-1 antibody (7C7, one of self- developed anti-MIC-1 high-affinity antibodies). Meanwhile, 50 μl 0.2 μg/ml of biotinylated rabbit anti-MIC-1 polyclonal antibody was added and incubated for 1 h at 37°C. After the plate was washed, streptavidin-HRP conjugate was added and incubated for 0.5 h at 37°C. Finally, the optical density of each well was determined using a microplate reader set to 450 nm. All samples were assayed in duplicate. Serum level of CEA and CA19.9 were detected by the related kit (Roche). Serum level of CA242 was detected by CA242 assay kit (Abbott).

### Statistical analysis

The Kruskal–Wallis test and Mann–Whitney test were used to compare the level of MIC-1 among all groups and between unpaired groups, respectively. The Wilcoxon test was used to compare MIC-1 level in paired serum samples obtained before surgical tumor resection and one month after surgical tumor resection as well as at the time of recurrence. Spearman bivariate correlation analysis was used to analyze the correlation. Receiver operating characteristic (ROC) analysis was performed to determine the diagnostic performance. Logistic regression model was also fitted to combine diagnostic information of biomarkers. The level of MIC-1 mRNA in tissues and protein in serum were described as mean ± SEM and mean ± standard deviation, respectively. The statistical analyses were performed with the statistical package for the social sciences, version 13.0 (SPSS), and a two-sided P value less than 0.05 was considered to be statistically significant.

## Results

### The overexpression of MIC-1 in PDAC tissues and the correlation with serum MIC-1

**Figure 1:**
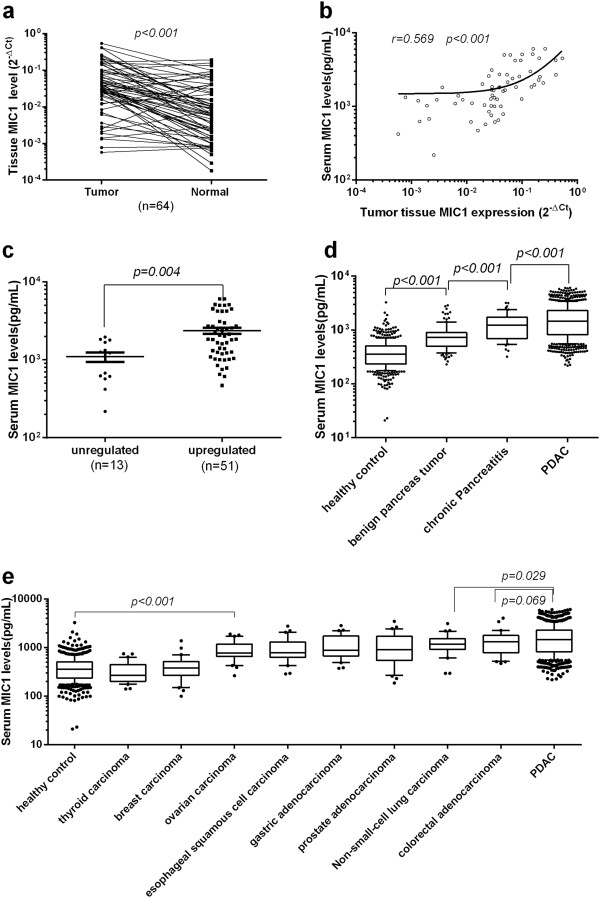
**The level of MIC-1 in PDAC tissue and serum samples. a**. Upregulation of MIC-1 in tumor tissues and corresponding normal samples in PDAC (y-axis: the MIC-1 mRNA expression level, described as 2-△Ct with log10 scale axis). **b**. Scatter plots showing the correlation between relative expression of MIC-1 levels in serum (y-axis: log10 scale) and matched tumor tissues (x-axis: log 10 scale) obtained from 64 patients. A positive correlation was found by Spearman correlation (r = 0.569; P < 0.001). **c**. Relationship between serum MIC-1 and MIC-1 overexpression in 64 patients with PDAC. **d**. Comparison of serum MIC-1 levels between the patients with PDAC and different controls. Serum MIC-1 levels of patients with PDAC are significantly higher than that of different controls. **e**. Serum MIC-1 in other malignant neoplasms, including eight kinds of common tumors. In the box plots, the lines denote 10th, 25th, median, 75th and 90th percentiles for each, using the Mann–Whitney U test.

First, we assessed the expression of MIC-1 in PDAC, and found that MIC-1 was overexpressed in 81.0% (51/64) of cancer tissues compared with their corresponding normal tissues. Additionally, 75% of the 64 cases showed at least a 2-fold upregulation (Figure 1a). The results also showed that increased expression of MIC-1 was not significantly correlated with TNM classification (stage I: 0.063 ± 0.019, stage II: 0.087 ± 0.018; P = 0.172), suggesting that the overexpression of MIC-1 likely occurred in the early stages of PDAC.

Based on our previous result that MIC-1 acts a secretory protein, the correlation between MIC-1 protein expression levels in 64 paired PDAC tissues and matched serum samples was also analyzed. We observed a statistically significantly positive correlation between MIC-1 expression in tumor tissues (0.077 ± 0.013) and matched serum samples (2085.9 ± 1477.6 pg/mL) from these patients (r = 0.569, P < 0.001; Figure 1b), and the serum MIC-1 level in these patients with upregulated expression of MIC-1 in tumor tissues (n = 51) was significantly higher than that with down-regulated expression of MIC-1 (n = 13) (P =0.004; Figure 1c). Therefore, we focused the rest of our study on serum MIC-1 for further assessment of its efficacy as a diagnostic and prognostic biomarker in patients with PDAC.

### The elevated level of serum MIC-1 in PDAC and its diagnostic potential

To evaluate the diagnostic potential of MIC-1, a total of 1472 serum samples, including those from patients with PDAC (n = 807), benign pancreas tumor (n = 115), chronic Pancreatitis (n = 50) and normal control subjects (n = 500) were examined. In comparison with healthy control subjects (416.8 ± 286.9 pg/mL), the levels of serum MIC-1 demonstrated a stepwise increase in patients with benign pancreas tumor (808.4 ± 483.9 pg/mL; P < 0.001), chronic Pancreatitis (1299.0 ± 709.6 pg/mL; P < 0.001) and PDAC (1731.0 ± 1181.0 pg/mL; P < 0.001) (Figure 1d). The results also showed that increased expression of MIC-1 was not significantly correlated with TNM classification (P = 0.212, Kruskal–Wallis test), which was consistent with the result of MIC-1 expression in tissues, again suggesting that the increased level of serum MIC-1 might occur in early stage of PDAC.

To determine whether or not MIC-1 expression is unique for PDAC, we also collected serum from 240 individuals prior to surgery. This included samples from patients with eight different types of common epithelial malignancies (Table 2). Quantitative ELISA revealed that serum MIC-1 levels in PDAC were higher than in the eight other cancers tested. We also found that, when compared with normal controls (416.8 ± 286.9 pg/mL), serum MIC-1 was elevated in non-small-cell lung carcinoma (1258.0 ± 587.3 pg/mL; P < 0.001), gastric adenocarcinoma (1154.0 ± 660.2 pg/mL; P < 0.001), ovarian carcinoma (923.0 ± 442.7 pg/mL; P < 0.001), esophageal squamous cell carcinoma (1018.0 ± 618.7 pg/mL; P < 0.001), prostate adenocarcinoma (1167.0 ± 804.9 pg/mL; P < 0.001), and colorectal adenocarcinoma (1371.0 ± 818.7 pg/mL; P < 0.001); but there was no significant difference in levels of MIC-1 in thyroid carcinoma (336.4 ± 172.2 pg/mL; P = 0.132) and breast carcinoma (426.5 ± 264.0 pg/mL; P = 0.856) (Figure 1e).

### Better diagnostic performance of serum MIC-1 compared with CA199, CEA and CA242 in PDAC

**Figure 2:**
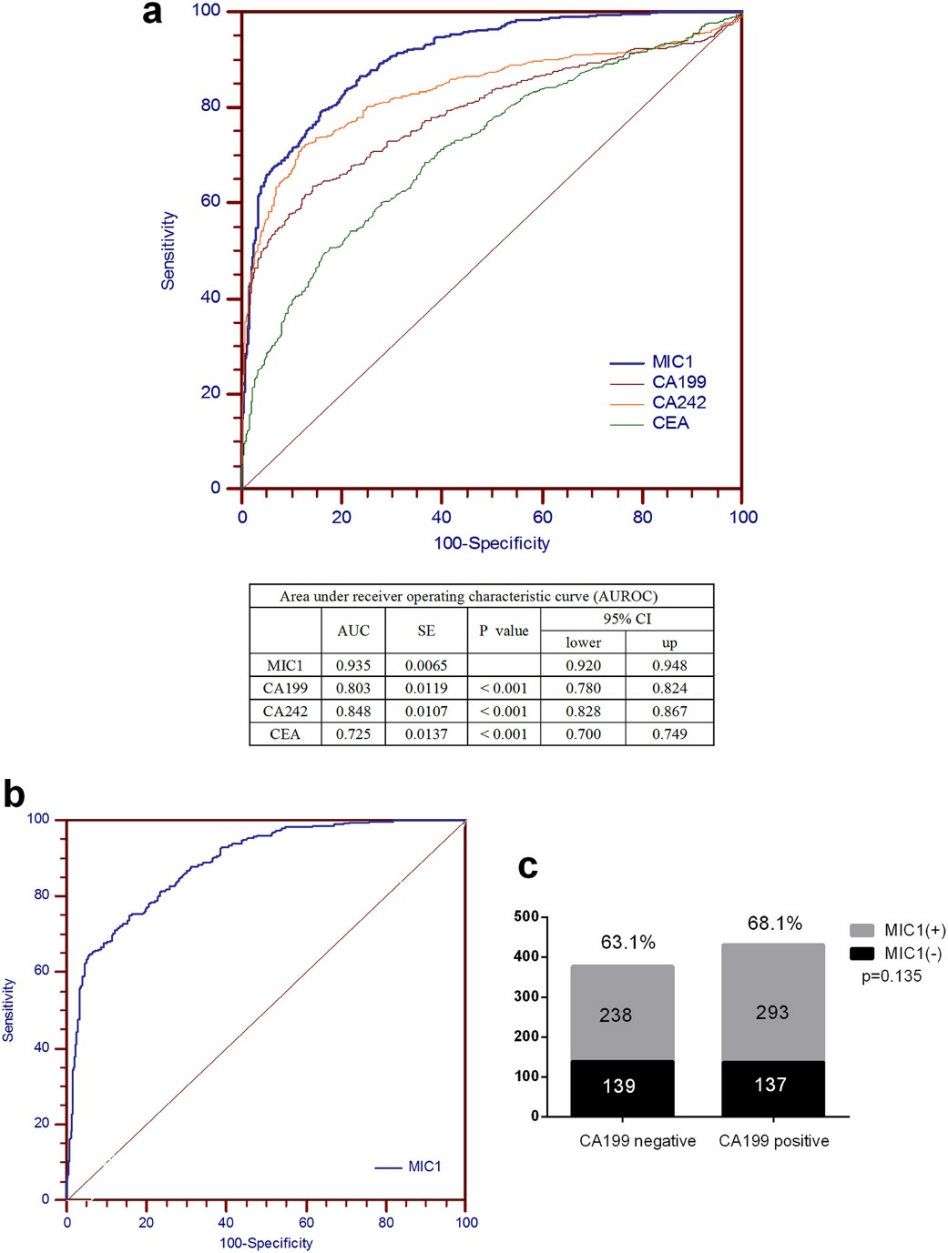
**Comparison of the diagnostic performance of serum MIC-1, CA19.9, CEA and CA242 for PDAC. a**. Sensitivities and specificities of MIC-1 , CA19.9, CEA and CA242 for the diagnosis of PDAC was compared through the analyses of ROC curves in the discovery group (n = 1307). AUROC curve of serum MIC-1 was much larger than that of CA19.9, CEA and CA242 (P < 0.001). **b**. The potential of serum MIC-1 for distinguishing CA19.9-negative pancreatic carcinomas from non–pancreatic carcinoma controls including benign pancreas tumors. **c**. A similar positive rate (present above the bar) of serum MIC-1 (using the cut off value 1000 pg/mL) was observed in patients with PDAC with different CA19.9 levels.

We next generated ROC curves to assess the potential usefulness of serum MIC-1 as a noninvasive biomarker for PDAC in the discovery group. Using the 500 normal samples as negative controls, the area under the ROC curve of MIC-1 for PDAC is higher than that of CA19.9, CEA and CA242 (P < 0.001; Figure 2a). Using a cutoff value of 1000 pg/ml, based on mean plus three standard deviations of healthy subjects for the sake of usability in clinical settings, the sensitivity, specificity, and positive and negative predictive values of MIC-1 were 65.8%, 96.4%, 96.7%, and 63.6%, respectively, to identify a patient with PDAC. The sensitivity of MIC-1 for diagnosis of PDAC was higher than that of CA19.9 (53.3%), CEA (29.6%) and CA242 (48.9%) and demonstrated comparable specificity. More importantly, the sensitivity of MIC-1 was independent of serum CA19.9 levels (r = 0.066, P = 0.061). Therefore, the diagnostic performance of serum MIC-1 was also carefully investigated in CA19.9-negative (<37 U/mL) PDAC. We noticed that serum MIC-1 had an outstanding performance for distinguishing CA19.9-negative pancreatic carcinomas from non–pancreatic carcinoma controls including benign pancreas tumors (AUROC, 0.886; 95% CI, 0.865–0.906; Figure 2b). These results suggested that serum MIC-1 level is a much more sensitive tumor marker compared to CA19.9 for the detection of pancreatic carcinomas. MIC-1 demonstrated superiority even in those PDAC with negative CA19.9 (<37 U/mL; n = 377), showing a median serum MIC-1 level of 1253.3 pg/mL and a sensitivity of 63.1% (Figure 2c). Moreover, multivariate logistic regression model indicated that the combination of MIC-1 and CA19.9 could improve the diagnostic performance significantly (AUROC, 0.957; 95% CI, 0.945 –0.967).

To explore the ability of MIC-1 as a single marker in discriminating patients with PDAC from benign disease, the control group involved subjects with chronic pancreatitis or benign pancreas tumors, respectively. Our ROC analyses revealed that serum MIC-1 as a single marker was insufficient to discriminate patients with PDAC from benign subjects, with an AUC value of 0.739, which was not found to be superior to serum CA242 (0.739), CA19.9 (0.520) and CEA (0.619). At the cutoff value of 1000 pg/mL, only 10.42% (12 of 115) of patients with benign pancreas tumor exceeded the threshold; in contrast, 8.7% (10 of 115), 15.7% (18 of 115) and 15.7% (18 of 115), of these patients were above the cutoff value of CA242, CA19.9 and CEA, respectively. Results also indicated that MIC-1 (AUC, 0.592) is inferior to CA19.9 (0.684), CEA (0.620) and CA242 (0.739) in the distinction of PDAC from chronic pancreatitis, which may be attributed to MIC-1’s association with inflammation.

### Performance of serum MIC-1 for the diagnosis of PDAC at early stage and validation in another independent cohort

**Figure 3:**
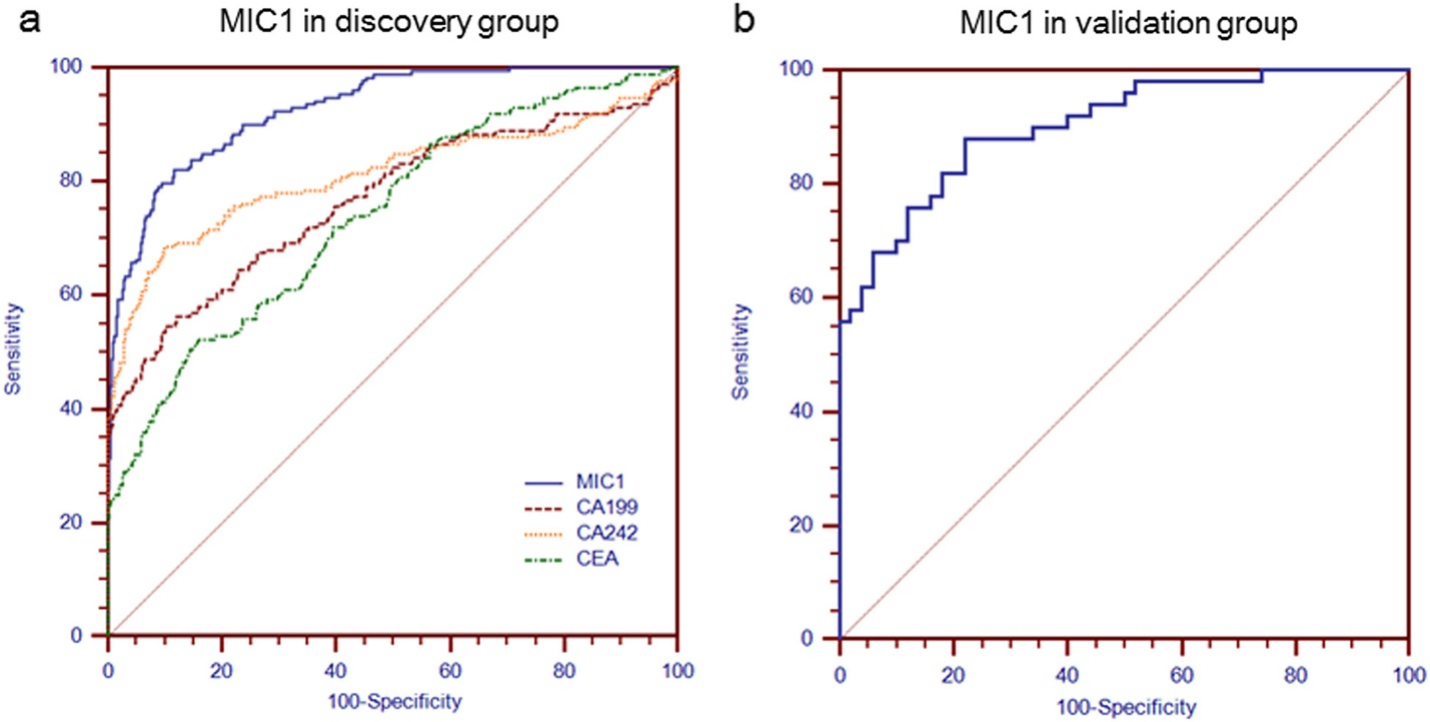
**The role of serum MIC-1 in the diagnosis of early stage PDAC. a**. ROC curve analysis using serum MIC-1, CA199, CEA and CA242 levels for discriminating PDAC in discovery group. **b**. ROC curve analysis using serum MIC-1 levels for discriminating PDAC in validation group.

To evaluate the diagnostic performance of MIC-1 in early detection and diagnosis of PDAC, we focused on a subset of patients with early-stage PDAC (stage I and II; n = 172). ROC curve analysis suggested that serum MIC-1 had a better performance compared with CA19.9, CA242 and CEA for distinguishing early-stage PDAC from normal controls (Figure 3a). In detecting early-stage pancreatic carcinomas (stage I and II), the sensitivity of MIC-1 was much higher than that of CA19.9 (65.1% vs.43.0%); even in very early-stage pancreatic carcinomas (stage Ia; n = 16), MIC-1 showed an obviously higher sensitivity of 62.5% compared to CA19.9’s sensitivity of 25.0%. In addition, the combination of CA19.9 and MIC-1 further significantly improved the detection rate of early PDAC (stage I and II) from 43.0% to 78.1%, which was much higher than the simultaneous use of CA19.9, CEA and CA242 (58.1%).

To further assess the robustness of the serum MIC-1 level as a novel early diagnostic marker in PDAC, we blindly validated in another external, independent group of 50 early-stage (stage I) PDAC and 50 healthy subjects (the validation group; Figure 3b). The serum MIC-1 level of PDAC (1357.0 ± 956.4 pg/mL; P < 0.001) was also significantly increased compared with that of healthy population (411.3 ± 190.5 pg/mL), which was quite similar to that of the early-stage PDAC derived from the discovery group.

### The roles of serum MIC-1 levels in monitoring treatment response of PDAC

**Figure 4:**
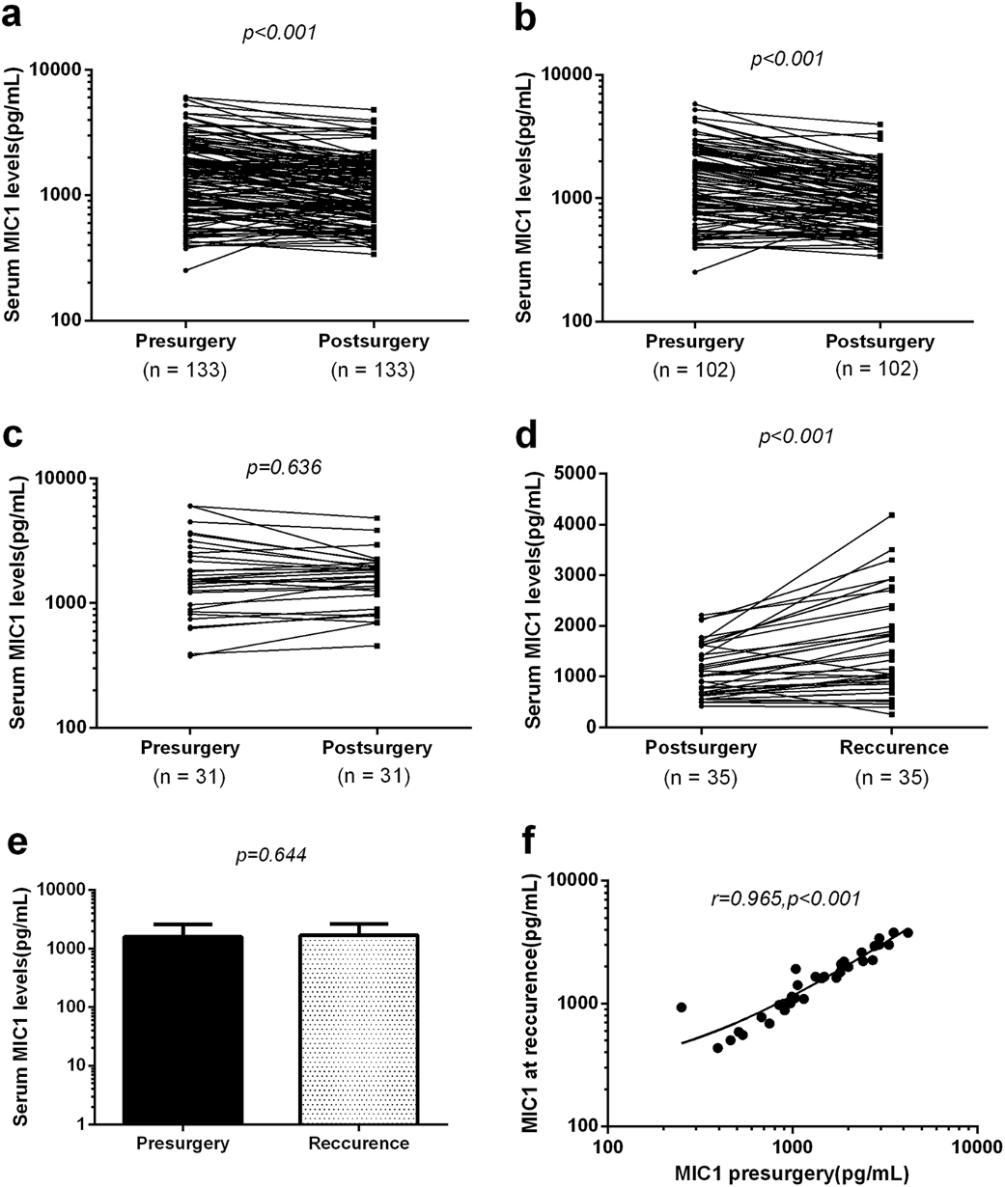
**The role of MIC-1 in evaluating therapy response and surveillance of PDAC after curative resection. a**. Comparison of serum MIC-1 levels from all PDAC patients before surgery (Pre) and one month after postsurgical removal of primary tumors (Post). (n = 133). **b**. Comparison of serum MIC-1 levels in 102 PDAC patients who underwent potentially curative surgeries. **c**. Comparison of serum MIC-1 levels in 31 PDAC patients who underwent noncurative surgeries. **d**. In 35 patients with documented recurrence, the serum MIC-1 levels were increased again to the preoperative levels. **e**. Comparison of serum MIC-1 levels between the baseline MIC-1 levels before the first operation and that at time of tumor recurrence in 35 patients with recurrence. **f**. A significant correlation (r = 0.965; P < 0.001) was found between MIC-1 levels before the first operation and after recurrence.

We analyzed paired pre- and postoperative serum samples in a subset of 133 PDAC patients who underwent surgical resection of their tumors. Among the 133 patients with PDAC, 102 underwent potentially curative resection, whereas 31 underwent noncurative resection. It was interesting to note that serum levels of MIC-1 (1728.0 ± 1218.0 pg/mL) were significantly reduced at one month after surgery (1280.0 ± 816.7 pg/mL; P < 0.001) (Figure 4a). Furthermore, when data was analyzed based on potentially curative vs noncurative surgeries, postoperative reductions in serum MIC-1 levels occurred exclusively among patients with potentially curative surgeries (1650.0 ± 1124.0 vs 1092.0 ± 635.9 pg/mL; P < 0.001) (Figure 4b). In contrast, no statistically significant differences were observed in MIC-1 levels before (1984.0 ± 1479.0 pg/mL) or after surgery (1726.0 ± 907.5 pg/mL) in patients with noncurative resections (P = 0.636) (Figure 4c). Collectively, these data underscore the importance of serum MIC-1 level as a potential biomarker for monitoring the treatment response of PDAC.

At one month after potentially curative resection, serum MIC-1 levels were significantly decreased from 1650.1 ± 1123.9 pg/mL to 1092.2 ± 635.9 pg/mL (P <0.001), a lower level similar to that of benign pancreas tumor. Moreover, in 35 cases with tumor relapse, the decreased serum MIC-1 levels after operation (1110.0 ± 515.6 pg/mL) were elevated again at the time of tumor recurrence (1710.0 ± 946.5 pg/mL; P <0.001) (Figure4d). A significant correlation (r = 0.965; P < 0.001) was found between the baseline MIC-1 levels before the first operation (1602.0 ± 998.4 pg/mL) and that at time of tumor recurrence (1710.0 ± 946.5 pg/mL; P = 0.644) (Figure 4e and f). These data suggested that MIC-1 could be a sensitive tumor marker to monitor the treatment response and post operation tumor recurrence in patients with PDAC.

## Discussion

Despite the variation of practice pattern worldwide, surgical resection is the current therapeutic option with curative intent for patients with PDAC [2, 5]. The 5-year survival rate after curative treatment for patients with early stage PDAC is more than 30%, whereas the 5-year survival rate for patients with advanced-stage disease remains very dismal [1, 3, 4]. Therefore, early detection and diagnosis of PDAC are extremely important in improving the survival of the patients. Currently, CA19.9 is still the only widely used serologic tumor marker in screening and diagnosing PDAC; however, the sensitivity and specificity is not satisfactory, especially for early stage PDAC [6, 7]. Although tremendous efforts have been applied to identify improved PDAC biomarkers such as CA242, to date, it has not been shown to be superior to CA19.9 in clinical performance [6, 8–10, 35]. Therefore, an additional biomarker favoring early detection and diagnosis of PDAC is still urgently needed. The present study is the first large-scale investigation of the clinical value of MIC-1 in PDAC. We analyzed the expression of MIC-1 in PDAC tissues and sera, and found that the serum levels of MIC-1 were elevated, which was consistent with the results observed in the tissue samples. Importantly, we also assessed the value of MIC-1 as a diagnostic indicator in early stage PDAC, and investigated the potential of serum MIC-1 for predicting response to therapies and tumor recurrence.

First, we found that the serum MIC-1 level of patients with PDAC is significantly higher than that of healthy controls, benign pancreas tumors and chronic pancreatitis populations, indicating that MIC-1 may serve as a promising biomarker in diagnosis of PDAC. Furthermore, we have explored the serum levels of MIC-1 in eight other common epithelial malignancies. The results indicate that serum MIC-1 levels are higher in PDAC patients than in patients with any of the other tumors (p = 0.067 compared with colorectal adenocarcinoma, each P < 0.05 compared with other seven tumor group), which implies that high levels of MIC-1 may serve as a promising biomarker in diagnosis of PDAC.

In our present study, we also compared the diagnostic value for PDAC of MIC-1 with CA19.9, CEA and CA242, which are often used as PDAC markers in clinical settings, further demonstrating that the diagnostic value of MIC-1 for PDAC was significantly better than the other three markers. More importantly, the elevation of serum MIC-1 was independent of CA19.9 level because a similar positive rate was observed when stratified by different serum CA19.9 status and no correlation between these 2 markers was found. Even in those CA19.9-negative PDAC, the serum MIC-1 level was also increased dramatically and the diagnostic sensitivity was 63.1%, which is much higher than that of the other reported biomarker such as serum CA242 and CEA in our study. The combination of MIC-1 and CA19.9 could improve the diagnostic performance significantly. These findings indicate that MIC-1 may become a novel diagnostic tumor marker to detect PDAC.

However, our ROC analyses revealed that serum MIC-1 levels were insufficient to discriminate patients with PDAC from benign pancreas tumors, with an AUC value of 0.739, although this was found to be superior to serum CA19.9 (0.520). At the cutoff value of 1000 pg/mL, 10.42% (12 of 115) of patients with benign pancreas tumors exceeded the threshold; in contrast, 15.7% (18 of 115) of these patients were above the cutoff value of CA19.9. These indicate that MIC-1 is a novel marker with a comparative false-positive rate in diagnosing and differentiating PDAC from benign pancreas tumors. In addition, ROC curves showed a lower classification power of MIC-1 with respect to CA19.9 among chronic pancreatitis and PDAC, which may be attributed to MIC-1’s association with inflammation.

Another interesting finding of our study is that MIC-1 showed a superior diagnostic performance compared to CA19.9 in those PDAC with early stage disease (stage I and II; sensitivity, 65.1% vs.43.0%). Even in those patients with very early stage disease (stage Ia), MIC-1 showed a much higher sensitivity of 62.5% compared to 25.0% in CA19.9. In addition, the combination of CA19.9 and MIC-1 further significantly improved the detection rate of very early PDAC from 43.0% to 78.1%, which was much higher than the simultaneous use of CA19.9, CEA and CA242 (58.1%). Thus, the combination of MIC-1 and CA19.9 may be a promising strategy for early diagnosis of PDAC in the future. Additionally, we preliminarily tested the serum MIC-1 in another independent cohort of early stage PDAC and found it was significantly elevated compared to the controls (P = 0.001). It is undeniable that there are many differences in the patient populations studied for each marker and combination of markers. However, these results strongly indicate that MIC-1 may serve as a more valuable tumor marker than CA19.9 in early detection of PDAC.

Monitoring response to therapies and tumor recurrence is another important role of tumor markers. In our present study, radical resection of PDAC resulted in a significant reduction in serum MIC-1 to a lower level that was similar to benign pancreas tumors, and the decreased serum MIC-1 was increased again at the time of tumor recurrence. This led us to hypothesize that serum MIC-1 may possibly play a role in the tumorigenesis and progression of PDAC. Moreover, the results suggested that monitoring of serum MIC-1 after surgery is useful in evaluation of early recurrence. These findings need to be explored further, as our sample size and the duration of follow-up are limited for this analysis; however, these findings do provide preliminary evidence of a relationship between serum MIC-1 levels and MIC-1 recurrence that warrants additional investigation. Moreover, most of these PDAC were treated by non-curative surgery or other curative therapies without detailed clinical data; therefore, the association between MIC-1 level and survival was not analyzed in this study and will need to be further investigated [36].

Although our current MIC-1 assay may become a promising tool for PDAC, we acknowledge potential limitation of using MIC-1 as a single biomarker for the early detection of PDAC. Circulating serum MIC-1 has been described in many solid cancers besides PDAC, underscoring the need for being vigilant about organ and disease specificity while investigating MIC-1 as solitary biomarker for PDAC [33, 37–40]. As a consequence, it might be challenging to differentiate whether circulating MIC-1 is specifically associated with PDAC itself or if this is a common phenomenon that manifests during progression of any cancer as a result of perturbations in the host immune response [14, 41]. Another limitation, as indicated above, was the fact that MIC-1 alone may not be sufficient to distinguish PDAC from chronic pancreatitis.

## Conclusions

In summary, our results provide compelling evidence for the potential usefulness of serum MIC-1 as an additional noninvasive diagnostic and therapeutic monitoring marker in patients with PDAC, a concept that can be incorporated into routine clinical practice in the not-so-distant future pending validation in large-scale multicenter prospective trials.
